# Hourglassing of Prolapsed Membranes With Fetal Parts Within 22 Weeks Primigravida: A Rare Presentation

**DOI:** 10.7759/cureus.30205

**Published:** 2022-10-11

**Authors:** Sainidhi G Reddy, Roohi Gupta, Rajasbala Dhande, Vadlamudi Nagendra, Srinidhi Cherukuri

**Affiliations:** 1 Radiodiagnosis, Jawaharlal Nehru Medical College, Datta Meghe Institute of Medical Sciences, Wardha, IND; 2 Obstetrics and Gynaecology, Jawaharlal Nehru Medical College, Datta Meghe Institute of Medical Sciences, Wardha, IND

**Keywords:** preterm birth, bleeding per vagina, prolapse of fetal membranes and parts, cervical incompetence, ultrasound

## Abstract

Three clinical scenarios, premature labor; inescapable abortion; and incompetent cervix, result in the dilatation of endocervical canals before term. Ultrasonography is the modality of choice for confirming the above conditions. Here, we discuss a case of preterm primigravida with complaints of bleeding per vagina with the dilated cervix and prolapsed membranes with fetal parts within.

## Introduction

Prolapsed membranes indicate premature birth, especially in the absence of immediate surgical intervention, and exposure of the membranes to the vaginal flora may predispose them to infection or rupture [1-3]. With particularly high rates of illness and death at extremely early gestations, preterm birth continues to be a leading cause of newborn morbidity and mortality. At 25 weeks of gestation, the predicted survival rates are 54%, at 24 weeks, 38%, and at 23 weeks, 23% [4]. The morbidity for infants born between 24 and 26 weeks of gestation remains high despite advances in neonatal care; around 50% of infants born before 25 full weeks have one or more disabilities [5].

According to studies, a short cervix accurately predicts preterm birth, whereas an open cervix with prolapsed membranes in the middle of the third trimester signifies a graver prognosis [6,7]. Before alterations can be seen digitally or by a speculum examination, ultrasonography can detect early membrane herniation in a cervical orifice (os) that is still intact [8].

## Case presentation

A 22-year-old primigravida presents to the ER department with complaints of bleeding per vagina and pain in the abdomen. On general examination, the patient was stable with normal blood pressure and pulse rate. The patient gave a history of lifting heavy weights, suggesting trauma. Per abdomen, she was 20 weeks pregnant with a normal fetal heart rate, adequate fetal movements, and was bleeding per vaginal. She was then shifted to the department of radiology for an emergency ultrasound.

**Figure 1:**
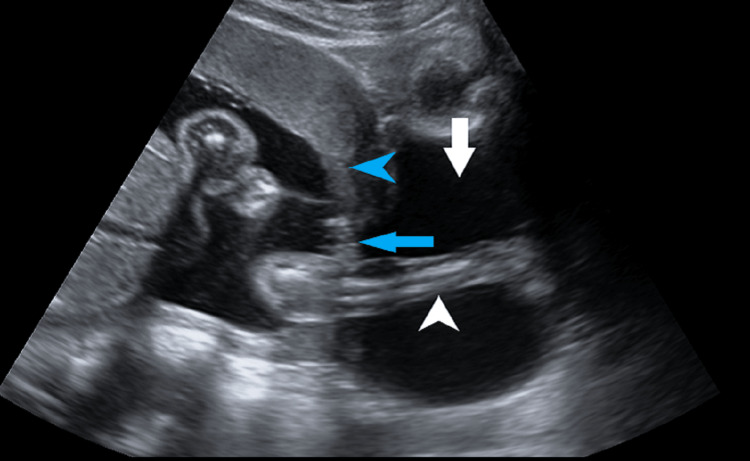
Transabdominal ultrasound image of the sagittal section showing an open U-shaped internal os (blue arrow) and a dilated cervix (white arrow) with fetal membranes and parts (feet) within (white arrowhead). The placenta is anterior, grade 1, with a lower margin reaching up to the internal os (blue arrowhead) suggesting placenta previa grade 3. os: orifice.

On ultrasound, the imaging findings were U-shaped prolapsed fetal membranes with fetal parts within, giving the typical hourglassing of prolapsed membranes through the open internal os and dilated cervix (Figure 1).

**Figure 2:**
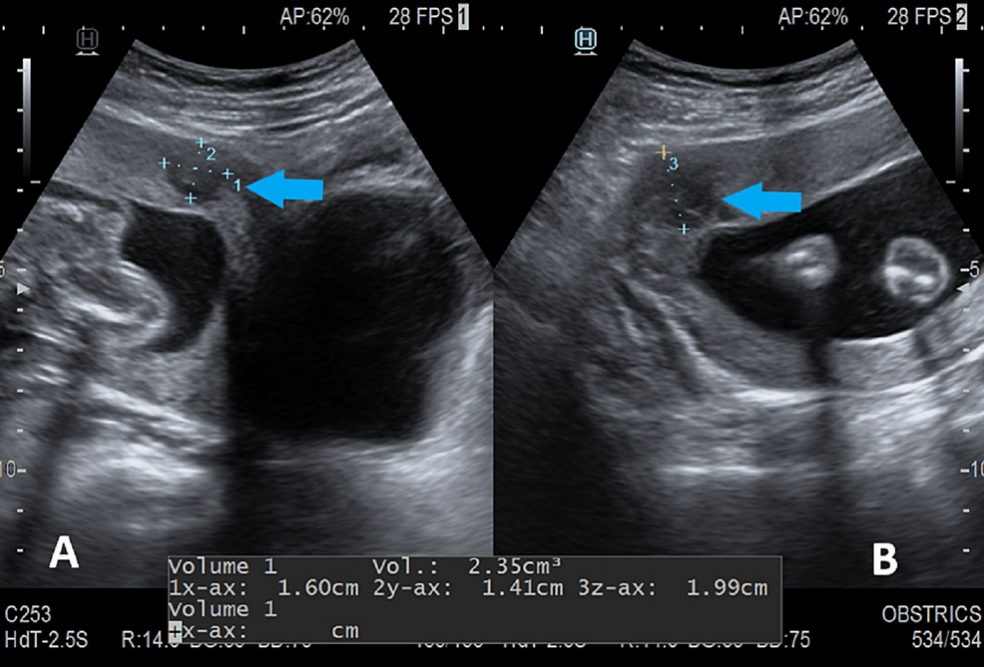
Transabdominal ultrasound sagittal (A) and axial (B) sections show minimal placental separation with a small retroplacental collection measuring approximately 2.35cc (blue arrow) suggesting retroplacental hemorrhage.

The lower end of the placenta reached up to the internal os (placenta previa grade 3) with mild placental separation at the lower back and retroplacental hematoma measuring up to 2.3cc (Figure 2).

**Figure 3:**
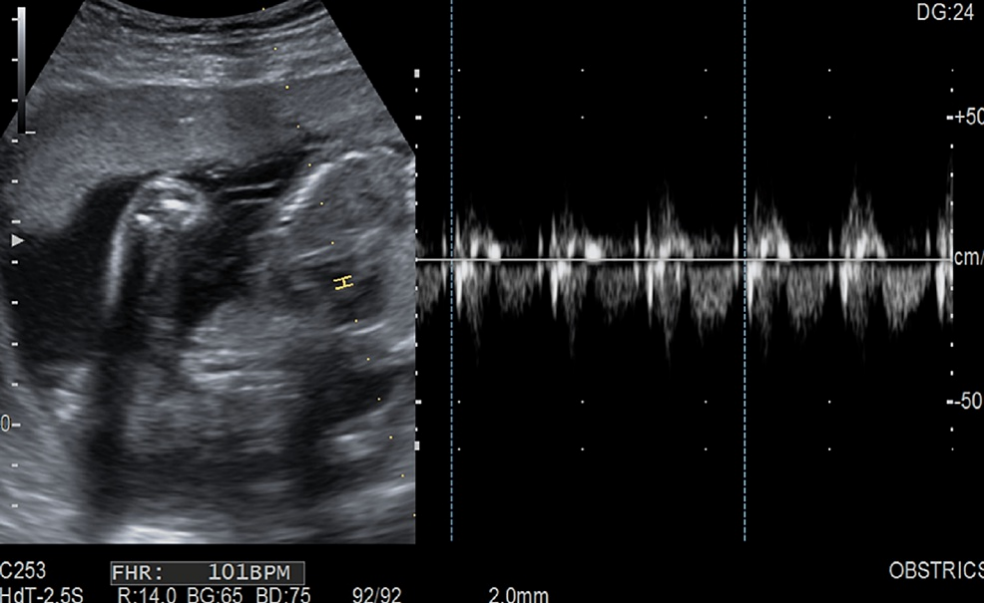
Transabdominal ultrasound shows an intrauterine live fetus with a fetal heart rate of 101 bpm. bpm: beats per minute.

Fetal movements were present, and the fetal heart rate was normal with adequate liquor (Figure 3). There was no history of documentation of a short cervix in the previous scans.

## Discussion

Cervical incompetence's cause is often unknown and is the topic of much conjecture. Possible causes include traumatic damage, congenital defects, and insufficient or defective collagen or elastin [9,10]. In 2002, Althuisius et al. proposed that cervical incompetence could manifest itself in a wide range of ways depending on the pregnancy, like preterm premature rupture of membranes, repeated abortions, or preterm labor [11]. Cervical insufficiency likely occurs in the middle of the second trimester or early in the third trimester, depending on the severity of the insufficiency. An incompetent cervix can occur at birth or develop over time. A failure in the embryological development of Mullerian ducts is the most frequent congenital cause. Due to a collagen shortage, the cervix cannot function as it should in conditions such as Ehlers-Danlos syndrome or Marfan syndrome, which result in insufficiency. The most frequent acquired cause is cervical trauma, which includes forced cervical dilatation during uterine evacuation in the first or second trimester of pregnancy, cervical conization, loop electrosurgical excision treatment (LEEP), and cervical lacerations sustained after birth. The majority of patients, however, experience cervical alterations as a result of infection or inflammation, which prompts early activation of the parturition pathway [12].

The ability to visualize the cervix via the abdomen has aided in the identification of cervical incompetence. In the chosen demographic of high-risk women based on an obstetrical history of a past spontaneous preterm birth, cervical ultrasonography has evolved as a validated, clinically helpful screening and diagnostic tool. Transvaginal ultrasounds frequently reveal a short cervical length (≤25 mm) or funneling, which is the ballooning of the membranes into a dilated internal os but with the external os closed [13]. The progression of a prolapsed fetal membrane starts with a long and closed cervix (T-shaped), to a Y-shaped, then a V-shaped, later evolving into a U-shaped funnel [14].

## Conclusions

Cervical incompetence is notoriously difficult to diagnose. The clinical evaluation alone is not accurate. Transabdominal ultrasonography plays an important role, which helps in the accurate diagnosis of cervical incompetence. Early diagnosis and management is the priority in cases of cervical incompetence, which reduces maternal and fetal morbidity and mortality rates.
